# A Novel Procedure for Precise Quantification of *Schistosoma japonicum* Eggs in Bovine Feces

**DOI:** 10.1371/journal.pntd.0001885

**Published:** 2012-11-15

**Authors:** Bin Xu, Catherine A. Gordon, Wei Hu, Donald P. McManus, Hong-Gen Chen, Darren J. Gray, Chuan Ju, Xiao-Jun Zeng, Geoffrey N. Gobert, Jun Ge, Wei-Ming Lan, Shu-Ying Xie, Wei-Sheng Jiang, Allen G. Ross, Luz P. Acosta, Remigio Olveda, Zheng Feng

**Affiliations:** 1 National Institute of Parasitic Diseases, Chinese Center for Disease Control and Prevention, Shanghai, People's Republic of China; 2 Molecular Parasitology Laboratory, Queensland Institute of Medical Research, Brisbane, Australia; 3 Infectious Disease Epidemiology Unit, School of Population Health, University of Queensland, Brisbane, Australia; 4 Jiangxi Provincial Institute of Parasitic Diseases Control, Nanchang, People's Republic of China; 5 Griffith Health Institute, Griffith University, Gold Coast, Australia; 6 Research Institute of Tropical Medicine, Department of Immunology, Manila, Philippines; Fundacao Oswaldo Cruz, Centro de Pesquisas Rene Rachou, Brazil

## Abstract

Schistosomiasis japonica is a zoonosis with a number of mammalian species acting as reservoir hosts, including water buffaloes which can contribute up to 75% to human transmission in the People's Republic of China. Determining prevalence and intensity of *Schistosoma japonicum* in mammalian hosts is important for calculating transmission rates and determining environmental contamination. A new procedure, the formalin–ethyl acetate sedimentation-digestion (FEA–SD) technique, for increased visualization of *S. japonicum* eggs in bovine feces, is described that is an effective technique for identifying and quantifying *S. japonicum* eggs in fecal samples from naturally infected Chinese water buffaloes and from carabao (water buffalo) in the Philippines. The procedure involves filtration, sedimentation, potassium hydroxide digestion and centrifugation steps prior to microscopy. Bulk debris, including the dense cellulosic material present in bovine feces, often obscures schistosome eggs with the result that prevalence and infection intensity based on direct visualization cannot be made accurately. This technique removes nearly 70% of debris from the fecal samples and renders the remaining debris translucent. It allows improved microscopic visualization of *S. japonicum* eggs and provides an accurate quantitative method for the estimation of infection in bovines and other ruminant reservoir hosts. We show that the FEA-SD technique could be of considerable value if applied as a surveillance tool for animal reservoirs of *S. japonicum*, particularly in areas with low to high infection intensity, or where, following control efforts, there is suspected elimination of schistosomiasis japonica.

## Author Summary

Schistosomiasis japonica, a chronic human parasitic disease in the People's Republic of China, the Philippines and areas of Indonesia, is a zoonosis with over 40 different mammals, including a number of ruminants, that can act as reservoir hosts for the infection. Precise identification of the major infection reservoirs is important for the control of *Schistosoma japonicum* as their targeted treatment can prevent environmental contamination and transmission of the parasite, thus reducing the risk to humans. Current copro-parasitological techniques are generally unsatisfactory for identifying and quantifying *S. japonicum* eggs in ruminant feces due to the large volume of cellulosic debris present. The new approach we describe here, the FEA–SD technique, removes much of this material by sieving and centrifugation with ethyl actate and renders any remaining debris transparent by use of a potassium hydroxide (KOH) digestion, providing much improved visualization of eggs, enabling the collection of more accurate data on *S. japonicum* infection in ruminants. This new tool will be of particular value for monitoring schistosome prevalence and intensity in animal reservoirs in areas of the People's Republic of China that are heading toward schistosomiasis elimination.

## Introduction

*Schistosoma japonicum*, the causative agent of Asian schistosomiasis, is endemic to the People's Republic of China, the Philippines and small pockets of Indonesia [1]–[7]. Unlike African schistosomiasis, mainly caused by *S. mansoni* and *S. haematobium*, schistosomiasis japonica is a zoonosis and it is estimated that over 40 mammalian species, comprising 28 genera and 7 orders of wild and domestic animals, can act as reservoirs and harbour *S. japonicum* infection [8]. The range of mammalian hosts complicates schistosomiasis control efforts and, as well as the public health considerations, the disease adds to the economic burden of communities as *S. japonicum* infection debilitates domestic livestock that are used for food and as work animals [9], [10]. Bovines, particularly water buffaloes (*Bubalus bubalis*), have been shown to be major reservoir hosts for schistosomiasis japonica in the lake areas and marshlands of southern the People's Republic of China [11]–[16]. However, their role in schistosome transmission has yet to be fully determined in other endemic areas, notably the Philippines, due partly to inconsistent results obtained with the different methods used for identifying and quantifying *S. japonicum* eggs in mammalian hosts [17]–[20]. In particular, the presence of bulk debris, including cellulosic fibrous material, in the feces of ruminants often obscures the eggs and impairs their visualization across all current copro-parasitological methods that involve microscopy.

Here we describe a new copro-parasitological method, the formalin–ethyl acetate sedimentation-digestion (FEA–SD) technique, which eliminates much of this bulk debris and cellulose material and facilitates much improved microscopic examination of *S. japonicum* eggs in the feces of bovines and other ruminant hosts. We show the FEA-SD technique is an effective technique for identifying *S. japonicum* eggs using fecal samples from naturally infected Chinese water buffaloes (*Bubalis bubalis*) and confirm its reproducibility using parasite-positive samples obtained from carabao, a subspecies (*Bubalus bubalis carabanesis*), in the Philippines. We show that the FEA-SD method is as efficient as real-time PCR (qPCR) for determining schistosome prevalence in bovines but is less costly to implement.

## Materials and Methods

### Ethics Statement

The conducts and procedures involving animal experiments were approved by the Animals Ethics Committee of the Queensland Institute of Medical Research (project no. P288). This study was performed in accordance with the recommendations of the Australian code of practice for the care and use of animals for scientific purposes, 2004.

### The FEA-SD Technique

**Figure 1:**
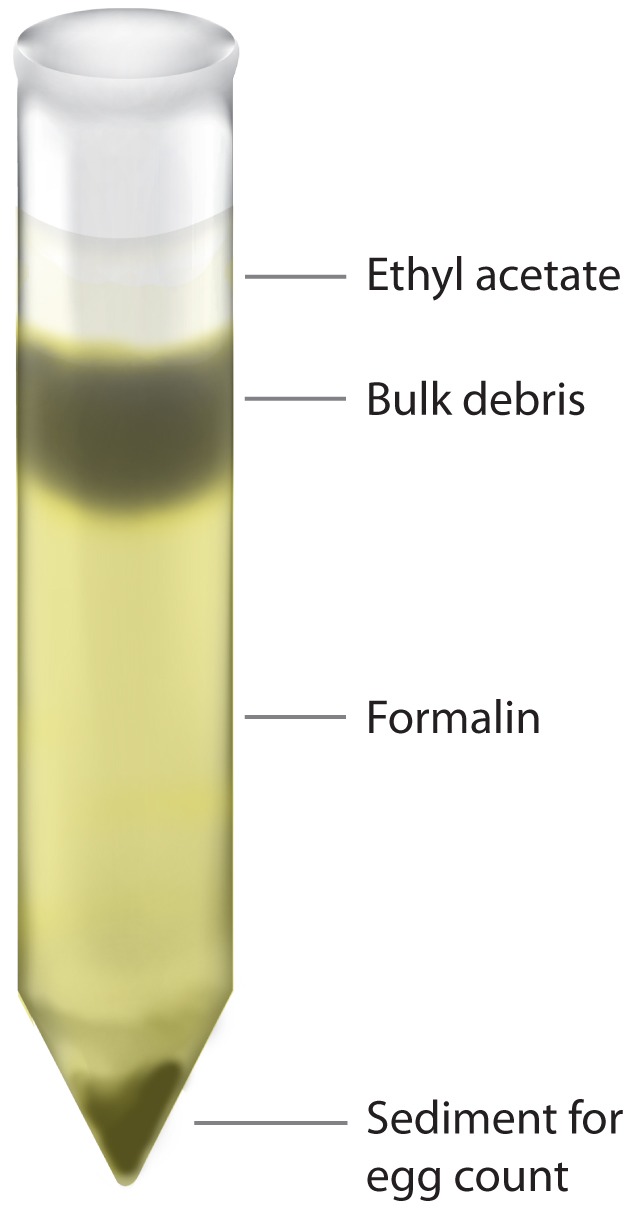
Four layer separation of sieved fecal material using the FEA-SD technique. The resulting four layer separation occurs after the addition of 100% ethyl acetate and centrifugation at 500 *g*. The top layer contains the ethyl acetate, the second layer the bulk debris to be discarded, the third layer comprises 10% (v/v) formalin and the final layer is the remaining sediment containing the eggs.

Stool samples were taken intra-rectally from 13 water buffaloes, collected from Jiangxi province, People's Republic of China, shown to be naturally infected with *S. japonicum*, by the miracidial hatching test (MHT) [21]. The FEA–SD technique was then used on the positive samples to identify *S. japonicum* eggs, calculating egg recovery rates and bulk debris reduction. Full details of the procedure are as follows: First, bovine stool samples are collected rectally from the animals (approximately 500 g each) by a veterinarian or trained personnel. Each stool sample is homogenized with an applicator stick and 50 g of the stool mixture is taken and mixed to a slurry in a beaker with 300 ml of water. The slurry is then sieved, by pouring the slurry onto a 60 copper mesh (Tyler scale with a pore opening size of 250 µm) and using water to flush the smaller sediment onto a 260 copper mesh (61 µm), held below the 40–60 mesh. Sediment caught on the 260 mesh is washed with water into a conical flask and allowed to sediment naturally for 30 minutes. The excess water is removed, leaving sediment which is poured into a 50 ml tube. Approximately 50 ml of water is added to the conical flask and naturally sedimented for 30 minutes, again removing excess water and pouring sediment into the same 50 ml tube. This is repeated once more, to ensure all sediment is in the 50 ml tube. The tube is topped up to 50 ml with 10% formalin (v/v) and mixed thoroughly by vortexing, before standing at room temperature for 30 minutes to fix the eggs. The Falcon tube is then vortexed and, using a pasteur pipette, 10 ml of the suspension (equivalent to 10 g feces) placed into two 15 ml tubes (5 ml in each; equivalent to 5 g feces) labelled A and B. Ten percent (v/v) formalin solution is added to tubes A and B to take the volume up to 8 ml and the tubes mixed thoroughly by vortexing, after which 4 ml of 100% (v/v) ethyl acetate is added using a glass pipette and vigorously vortexed once more for 30 sec. With the cap of the tubes slightly loosened the tubes are centrifuged at 500 *g* for 10 minutes, resulting in a four-layer separation (Figure 1). It is important for this step that the tubes are spun at 500 *g* for stable and efficient debris removal. The ethyl acetate is removed from both tubes by gently rimming the bulk debris layer with a thin applicator stick and decanting the top three layers which are then discarded. Ethyl acetate and 10% (v/v) formalin can be added and spun again if necessary to remove further bulk debris. If the middle layer of bulk debris is very thin the tube should be shaken vigorously and the re-spun for better efficiency. Water is added to the remaining pellet to take the volume up to 5 ml and an equal volume of 10% (w/v) potassium hydroxide (KOH) is added to each tube. The tubes are mixed gently by vortexing to resuspend the pellet and the sample is digested overnight at 37°C.

After digestion, the sample is vortexed vigorously and centrifuged at 900 *g* for 10 minutes. The pellet is washed once with 10–15 ml of water to remove any residual KOH by centrifuging the solution for 10 minutes at 900 *g*, the supernatant removed and the final pellet is resuspended in 4–6 ml of water (as the samples are now fixed) and stored at 4°C. The sample is now ready for counting the *S. japonicum* eggs by microscopy. Before counting, the suspension is mixed gently with a pipette and the total volume of tubes A and B counted for each sample, pipetting 200–300 µl onto each slide for microscopy. It is important that this is done as the sensitivity of the procedure increases with the amount of suspension examined. The microscopy was performed blind by two independent microscopists, although this is not essential to the completion of the procedure. Infection intensity (eggs per gram of feces, EPG) is calculated based on the total egg number in 10 g of feces (i.e., the contents of tube A plus tube B).

### Reduction in Bulk Debris and Egg Recovery

In order to determine the effectiveness and reproducibility of the FEA-SD technique in reducing the bulk debris and cellulosic material present in water buffalo feces, stool samples from the 13 Chinese animals were individually processed and the initial and final volumes of debris measured and compared (Table 1). Egg recovery was measured by microscopic examination of each of the normally discarded top 3 layers (Figure 1) for the presence of eggs (Table 2). Eggs in the final sediment were counted as per the protocol described above (Table 3).

**Table 1:** Reduction in bulk debris of water buffalo fecal samples from the People's Republic of China by the FEA-SD method.

	Volume of sediment (ml)			
*Sample	Initial (I)	Post FEA-SD (P)	I-P (ml)	I - P/I %
1A	1.3	0.2	1.1	84.6
1B	1.5	0.2	1.3	86.7
2A	1.6	0.6	1.0	62.5
2B	1.6	0.6	1.0	62.5
3A	1.0	0.4	0.6	60.0
3B	1.0	0.4	0.6	60.0
4A	1.7	0.3	1.4	82.4
4B	1.5	0.2	1.3	86.7
5A	1.8	0.6	1.2	66.7
5B	1.9	0.8	1.1	57.9
6A	2.0	0.8	1.2	60.0
6B	1.7	0.8	0.9	52.9
7A	1.3	0.4	0.9	69.2
7B	1.4	0.4	1.0	71.4
8A	2.5	0.8	1.7	68.0
8B	2.4	1.0	1.4	58.3
9A	1.4	0.3	1.1	78.6
9B	1.5	0.3	1.2	80.0
10A	1.0	0.3	0.7	70.0
10B	1.0	0.2	0.8	80.0
11A	1.0	0.2	0.8	80.0
11B	1.0	0.2	0.8	80.0
12A	1.0	0.3	0.7	70.0
12B	1.0	0.2	0.8	80.0
13A	1.0	0.5	0.5	50.0
13B	1.0	0.6	0.4	40.0
Mean	1.4	0.4	1.0	69.2
SD				12.3

* Equivalent to 5 g feces/sample; n = 26.

**Table 2:** Reduction in bulk debris of carabao fecal samples from the Philippines by the FEA-SD method.

Sample	Initial (I)	Post FEA-SD (P)	I-P (ml)	I - P/I %
1A	1.0	0.2	0.8	80.0
1B	1.2	0.3	0.9	75.0
2A	1.0	0.5	0.5	50.0
2B	1.1	0.5	0.6	54.5
3A	1.3	0.5	0.8	61.5
3B	1.4	0.5	0.9	64.3
4A	1.3	0.3	1.0	76.9
4B	1.4	0.4	1.0	71.4
5A	1.4	0.4	1.0	71.4
5B	1.5	0.4	1.1	73.3
6A	1.3	0.4	0.9	69.2
6B	1.3	0.3	1.0	76.9
7A	1.2	0.6	0.6	50.0
7B	1.2	0.6	0.6	50.0
8A	1.3	0.4	0.9	69.2
8B	1.3	0.3	1.0	76.9
9A	1.1	0.5	0.6	54.5
9B	1.0	0.3	0.7	70.0
10A	1.0	0.3	0.7	70.0
10B	1.1	0.5	0.6	54.5
11A	0.9	0.4	0.5	55.6
11B	1.0	0.5	0.5	50.0
12A	1.0	0.5	0.5	50.0
12B	1.2	0.4	0.8	66.7
13A	1.5	0.6	0.9	60.0
13B	1.6	0.5	1.1	68.8
14A	1.6	0.9	0.7	43.8
14B	1.6	0.9	0.7	43.8
15A	1.2	0.5	0.7	58.3
15B	1.2	0.6	0.6	50.0
16A	0.8	0.3	0.5	62.5
16B	0.7	0.3	0.4	57.1
17A	1.0	0.5	0.5	50.0
17B	1.0	0.5	0.5	50.0
18A	1.0	0.4	0.6	60.0
18B	1.1	0.4	0.7	63.6
19A	0.9	0.5	0.4	44.4
19B	0.9	0.5	0.4	44.4
20A	1.0	0.4	0.6	60.0
20B	1.0	0.4	0.6	60.0
22A	1.1	0.3	0.8	72.7
22B	1.0	0.3	0.7	70.0
23A	1.0	0.3	0.7	70.0
23B	1.2	0.4	0.8	66.7
24A	1.3	0.4	0.9	69.2
24B	1.4	0.5	0.9	64.3
25A	1.0	0.4	0.6	60.0
25B	1.1	0.3	0.8	72.7
**Mean**	**1.2**	**0.4**	**0.7**	**61.8**
**SD**				**10.2**

**Table 3:** Reproducibility in recovery of *S. japonicum* eggs in buffalo fecal samples from the People's Republic of China using the FEA-SD technique.

	Number of eggs					
Sample/Tube	Water supernatant (W)	Bulk debris (BD)***	W+BD	Bottom sediment	Total	Egg recovery rate (%)****
*Sample 1						
**Tube A1	0	1	1	50	51	98.0
Tube B1	1	2	3	66	69	95.7
Sample 2						
Tube A2	0	2	2	46	48	95.8
Tube B2	0	5	5	66	71	93.0
Sample 3						
Tube A3	0	2	2	45	47	95.7
Tube B3	0	5	5	64	69	92.8
Mean						95.2
SD						2.0

* Equivalent to 10 g feces.

** Equivalent to 5 g feces.

*** BD: Sediment in the bulk debris layer (Figure 1) removed during the FEA-SD procedure.

**** Egg recovery rate (%) = number of eggs in the bottom sediment/total number of eggs in the sample ×100.

### Application and Reproducibility of the FEA-SD Method Using Fecal Samples Collected from Philippines Carabao

Twenty-five carabao fecal samples were collected intra-rectally from *S. japonicum*-endemic barangays (villages) from Western Samar province, the Philippines. These were subjected to the FEA-SD procedure applied earlier in China with the debris reduction measured (Table 2) and the final egg counts from Tubes A and B for each of the 25 samples determined (Table 4). Full details of the sample collection and methods used can be found in Gordon *et al.* 2012 [22].

**Table 4:** Recovery of *S. japonicum* eggs in carabao fecal samples from the Philippines using the FEA-SD technique.

Sample/Tube	Eggs recovered*	Total	Sample/Tube	Eggs recovered*	Total
Sample 1			Sample 14		
Tube A1	3		Tube A14	1	
Tube B1	6	9	Tube B14	2	3
Sample 2			Sample 15		
Tube A2	2		Tube A15	0	
Tube B2	1	3	Tube B15	0	0
Sample 3			Sample 16		
Tube A3	0		Tube A16	0	
Tube B3	1	1	Tube B16	2	2
Sample 4			Sample 17		
Tube A4	0		Tube A17	5	
Tube B4	8	8	Tube B17	0	5
Sample 5			Sample 18		
Tube A5	9		Tube A18	5	
Tube B5	11	20	Tube B18	7	12
Sample 6			Sample 19		
Tube A6	1		Tube A19	2	
Tube B6	1	2	Tube B19	2	4
Sample 7			Sample 20		
Tube A7	2		Tube A20	0	
Tube B7	1	3	Tube B20	1	1
Sample 8			Sample 21		
Tube A8	0		Tube A21	5	
Tube B8	2	2	Tube B21	6	11
Sample 9			Sample 22		
Tube A9	0		Tube A22	1	
Tube B9	0	0	Tube B22	3	4
Sample 10			Sample 23		
Tube A10	2		Tube A23	3	
Tube B10	7	9	Tube B23	7	10
Sample 11			Sample 24		
Tube A11	0		Tube A24	2	
Tube B11	4	4	Tube B24	6	8
Sample 12			Sample 25		
Tube A12	2		Tube A25	0	
Tube B12	0	2	Tube B25	3	3
Sample 13					
Tube A13	0				
Tube B13	4	4			

### Comparison of the FEA-SD Method with Other Fecal Examination Techniques

The FEA-SD was compared directly with other fecal examination techniques including Kato-Katz (KK), the MHT, a validated qPCR assay and conventional PCR on 44 fecal samples collected during the same survey in Western Samar province referred to above. Full details of the sample collection and methods used can be found in Gordon *et al.* 2012 [22].

## Results

**Figure 2:**
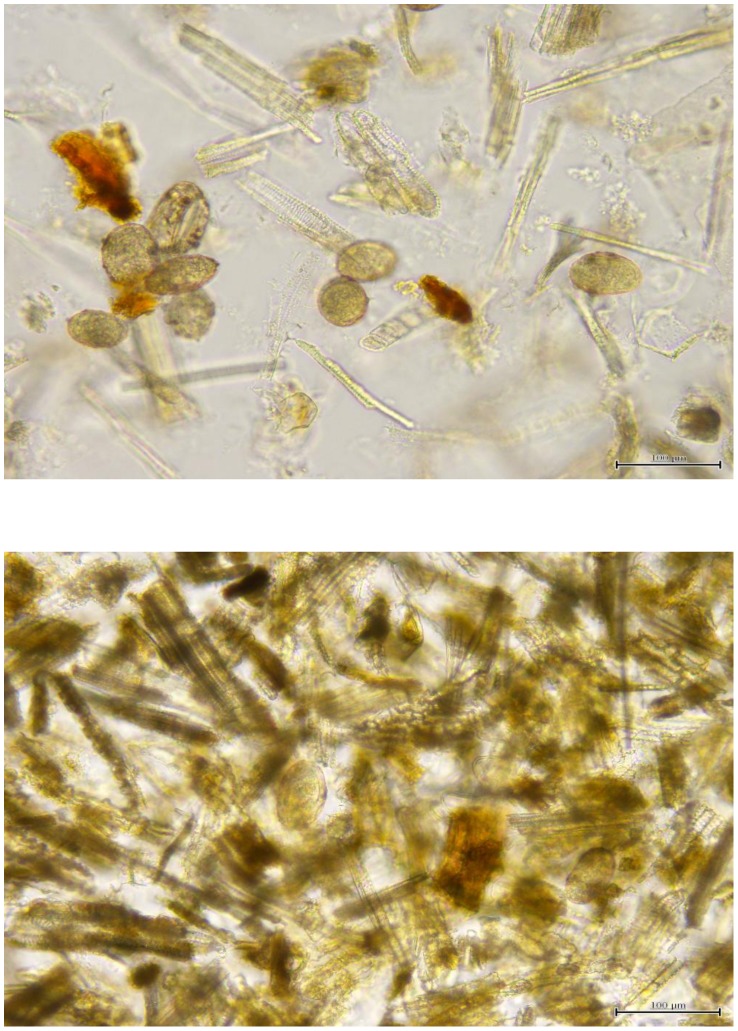
Visualization of *S. japonicum* eggs (circled) in water buffalo feces. Top panel; egg visualization after sieving of feces only. Lower panel; egg visualization after feces are subjected to the FEA-SD technique.

**Figure 3:**
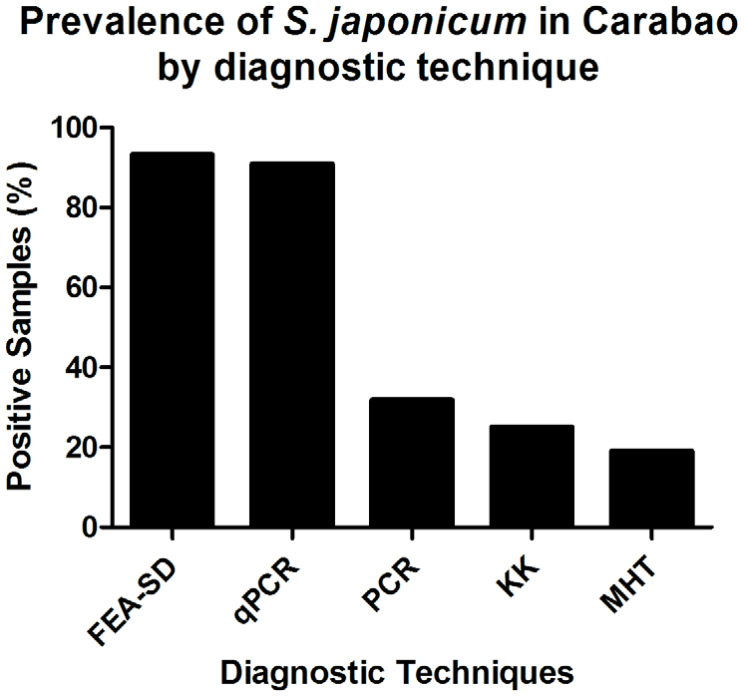
The FEA-SD method compared with other diagnostic techniques (Modified from Gordon et al. [22]). FEA-SD, formalin–ethyl acetate sedimentation-digestion technique; qPCR, quantitative PCR; PCR, conventional PCR; KK, Kato Katz method; MHT, miracidial hatching test. 95% confidence intervals are shown.

The FEA-SD technique removed an average of 61.5% — 69.2% of the bulk cellulose debris from the Philippine and Chinese, respectively, bovine stool samples prior to microscopic examination (Tables 1, 2). Any remaining debris was rendered transparent by the potassium hydroxide digestion step, so that eggs were readily observed compared with previous copro-parasitological techniques employing sieving only (Figure 2). Few eggs were present in the discarded bulk debris and supernatant, with an average of 95.2% of the total eggs recovered found in the final sedimented pellet (Table 3). Prevalence determined by the FEA-SD in the study undertaken on water buffaloes in the Philippines showed the FEA-SD (93.2%, 95% CI 85.4–100) had a similar sensitivity (90.9%, 95% CI 82.1–99.8) as the qPCR assay. By contrast the conventional PCR (31.8%, 95% CI 17.5–46.1), KK (25%, 95% CI 11.7–38.3) and MHT (19.1%, 95% CI 0.9–41.2) (Figure 3) gave much lower prevalence [21].

## Discussion

The amount of faeces excreted in one defecation by a large animal such as a water buffalo can exceed 45 kg so it is important to determine the minimal amount of feces that can provide optimal and consistent results using the FEA-SD technique. It was found that a sample of 10 g of feces (divided into tubes A and B) was critical for accurate quantification; as a comparison, 5 g of feces resulted in very inconsistent egg counts between tubes A and B.

The direct microscopic identification of schistosome eggs is the ‘gold’ standard for the diagnosis of zoonotic schistosomiasis in both animals and humans. The current microscopic methods of choice for the identification of *S. japonicum* eggs in bovines and other ruminants are, however, limited in terms of sensitivity and include, among other procedures, the MHT followed by a sedimentation filtration method, and the Danish Bilharziasis Laboratory (DBL) technique [23] (Table 5). More recently developed techniques include FLOTAC and the use of magnetic beads (Helmintex test) which have been shown to detect helminth eggs in low intensity infections [24]–[29]. Immunological techniques have also been applied to diagnostics however cross reactivity and identification of past infections, rather than current infections, have been issues. A recent study looking at Thioredoxin Peroxidase-1 in an ELISA system for identification of *S. japonicum* in bovines has shown promising results and no cross reaction with a closely related species [30].

**Table 5:** Published studies of diagnostic procedures for identification of *S. japonicum* eggs in bovine feces.

Location of study [Reference]	Year	Diagnostic	Bovine	Prevalence (%)	Intensity (EPG)
Poyang Lake, P. R. China [33]	2002	MHT+filtration	Cattle & water buffalo	14	42
Hubei, P. R. China [36]	1994	MHT+filtration	Water buffalo	35.7	0.4*
Anhui, P. R. China [37]	2010	MHT+filtration	Water buffalo	10.5	3.4
			Cattle	46.5	2.3
Sichuan, P. R. China [38]	2006	MHT+filtration	Cattle	22.3	-
Hunan and Jiangxi, P. R. China [12]	2007	MHT+filtration	Cattle	21.7	0.5–7.2*
			Water buffalo	14.9	0.5–7.2*
Dongting Lake, P. R. China [39]	2007	MHT	Cattle	6.1	-
			Water buffalo	9.5	-
Leyte, the Philippines [17]	2010	MHT	Water buffalo	0	-
		KK		3.7	-
		DBL Technique		3.7	-
		qPCR		51.5	2.1*
Samar, the Philippines [22]	2012	MHT	Water buffalo	19.1	-
		KK		25	4.7*
		FEA-SD		93.2	1.2*
		qPCR		90.9	6.1*
		PCR		31.3	-
Samar and Sorsogon, the Philippines [19]	2005	DBL technique	Water buffalo	6.3	-
Samar, the Philippines [40]	2007	DBL technique	Water buffalo	2.1	-
Mindoro, the Philippines [41]	1999	Formalin detergent technique	Water buffalo	0	-
Leyte, the Philippines [42]	1981	Merthiolate iodine-formaldehyde concentration (MIFC) technique	Cattle	0	-
			Water buffalo	0.38	-
Leyte, the Philippines [43]	1958	Glycerol sedimentation with egg hatching and sedimentation counting of eggs remaining	Water buffalo	1.5	-
			Cattle	3.82	-
Leyte, the Philippines [44]	1982	MIFC and Circumoval Precipitin Test (COPT)	Cattle	1 (MIFC)	-
			Cattle	0 (COPT)	-
			Water buffalo	9 (MIFC)	-
			Water buffalo	1 (COPT)	-
Cagayan, The Philippines [30]	2012	COPT ELISA	Water buffalo	34 (COPT)	-
				36 (ELISA)	-

* Geometric mean eggs per gram (EPG). All other values are arithmetic mean EPG.

MHT, miracidial hatching test; DBL, Danish Bilharziasis Laboratory method; KK, Kato-Katz technique; qPCR, quantitative real time PCR.

The MHT has been used extensively in the People's Republic of China for the identification of *S. japonicum* in bovine feces [11]. The MHT – a qualitative diagnostic test – involves the concentration of ova from saline using fresh feces through a nylon tissue bag and suspension in distilled water. Miracidia are visualized macroscopically and their presence is diagnostic of infection; three hatches (50 g feces per hatch) are routinely carried out. The MHT is preferred to the Kato Katz technique (KK) (recommended for diagnosis of intestinal schistosomiasis in humans) [26], [31], [32] and the other microscopic methods, due to the large volume of feces produced by bovines, and the fact that, as discussed, bovine feces contain considerable amounts of cellulosic material. This obscures the microscopic visualization of schistosome eggs making slide reading difficult and hindering diagnosis. A drawback of the MHT is that it has fairly rigid requirements for suitable pH, temperature and water quality, which cannot always be met under field conditions. Furthermore, it does not, on its own, provide infection intensity information and, like the KK, its sensitivity decreases as infection intensity decreases. In order to obtain intensity of infection estimates, additional microscopic visualization of eggs is performed on MHT-positive samples following a filtration sedimentation procedure whereby 50 g of feces are passed through 30 (595 µm) and 150 (90–105 µm) sieves and the flow through suspended in a nylon bag to capture the sediment which is then resuspended and the eggs present counted [33]. This is similar to the DBL technique [21] and the same problem of the presence of cellulosic material is common to both procedures.

The differences in sensitivity of these different techniques for examining ruminant feces for the presence of *S. japonicum* eggs makes it difficult to compare historical data for prevalence and incidence and infection intensity and to evaluate the involvement of potential reservoir hosts, particularly bovines in schistosomiasis transmission. Table 5 reviews the published studies of diagnostic procedures for the identification of *S. japonicum* in bovines and the inconsistent data obtained for prevalence and intensity. Telling examples are our recent pilot survey of *S. japonicum* infection in carabao from Western Samar [22] and the results of another recently published (2010) study on carabao from Leyte, the Philippines, which showed very low *S. japonicum* prevalence by KK (3.7%), the DBL technique (3.7%) and the MHT (0%) but a high prevalence (51.5%) using qPCR on the same fecal samples [17]. These two studies clearly highlighted the requirement for a more accurate microscopic technique, exemplified by the FEA-SD method, if only to validate diagnosis by qPCR.

The FEA-SD technique, including sieving, sedimentation, centrifugation and digestion, takes approximately 1.5 hours to complete. The length of time taken for subsequent slide reading depends on the skill and experience of the technicians involved, but two well trained and experienced microscopists are able to read one sample in 20 minutes. This procedure is relatively straight forward and only requires a centrifuge. Bovine feces comprise a large mass containing primarily cellulosic fibres and a direct count is the only way to get infection intensity but the debris obscure eggs to a large extent and the FEA-SD is the only currently available technique which clears a large proportion of the debris and renders remaining debris transparent, thereby increasing egg visualisation (Figure 2). Based on cost of reagents only, the FEA-SD technique is far less expensive ($US0.65) to perform than qPCR ($US9.2) although both approaches provide a very similar level of diagnostic accuracy [22]. We are currently using the FEA-SD method to determine the prevalence and intensity of *S. japonicum* in large animal cohorts as part of extensive epidemiological and surveillance studies we are undertaking in both the People's Republic of China and the Philippines.

In summary, the FEA-SD method is an improved tool that can be used to visualize schistosome eggs and to determine the prevalence and intensity of infection of *S. japonicum* in bovines. The increased visibility of eggs in the final sediment (Figure 2) compared with the DBL, MHT (+filtration) and KK techniques, makes the FEA-SD an important new technique applicable for epidemiological studies where bovines and other ruminants, such as goats, are potentially important reservoir hosts for *S. japonicum* [11]–[13], [34], [35]. In addition to *S. japonicum* the FEA-SD method can also be used to identify and quantify eggs of other helminths, such as *Fasciola* sp. in naturally infected animals. The FEA-SD also has the benefit of costing less than qPCR, which increases its potential as a surveillance tool for evaluating control programs, including in areas where control has led to the suspected elimination of schistosomiasis japonica.

## References

[1] Garjito TA, Sudomo M, Abdullah, Dahlan M, Nurwidayati A (2008) Schistosomiasis in Indonesia: past and present. Parasitol Int 57: 277–280. 18534900 10.1016/j.parint.2008.04.008

[2] Izhar A, Sinaga RM, Sudomo M, Wardiyo ND (2002) Recent situation of schistosomiasis in Indonesia. Acta Trop 82: 283–288. 12020902 10.1016/s0001-706x(02)00020-7

[3] Utzinger J, Zhou X, Chen M, Bergquist R (2005) Conquering schistosomiasis in China: the long march. Acta Trop 96: 69–96. 16312039 10.1016/j.actatropica.2005.08.004

[4] Wang L, Chen H, Guo J, Zeng X, Hong X, et al (2009) A strategy to control transmission of *Schistosoma japonicum* in China. N Engl J Med 360: 121–128. 19129526 10.1056/NEJMoa0800135

[5] Blas BL, Rosales MI, Lipayon IL, Yasuraoka K, Matsuda H, et al (2004) The schistosomiasis problem in the Philippines: a review. Parasitol Int 53: 127–134. 15081944 10.1016/j.parint.2004.01.003

[6] Leonardo LR, Rivera P, Saniel O, Villacorte E, Crisostomo B, et al (2008) Prevalence survey of schistosomiasis in Mindanao and the Visayas, the Philippines. Parasitol Int 57: 246–251. 18508406 10.1016/j.parint.2008.04.006

[7] McGarvey ST, Zhou XN, Willingham AL III, Feng Z, Olveda R (1998) The epidemiology and host-parasite relationships of *Schistosoma japonicum* in definitve hosts. Parasitol Today 15: 214–215. 10.1016/s0169-4758(99)01409-x 10366824

[8] He Y, Salafsky B, Ramaswamy K (2001) Host-parasite relationships of *Schistosoma japonicum* in mammalian hosts. Trends Parasitol 17: 320–324. 11423374 10.1016/s1471-4922(01)01904-3

[9] Blas BL, Lipayon IL, Tormis LC, Portillo LA, Hayashi M, et al (2006) An attempt to study the economic loss arising from *Schistosoma japonicum* infection and the benefits derived from treatment. Southeast Asian J Trop Med Public Health 37: 26–32. 16771209

[10] Ross AGP, Sleigh AC, Li Y, Davis GM, Williams G, et al (2001) Schistosomiasis in the People's Republic of China: prospects and challenges for the 21st century. Clin Microbiol Rev 14: 270–279. 11292639 10.1128/CMR.14.2.270-295.2001 PMC88974

[11] Gray DJ, Williams GM, Li Y, Chen H, Forsyth SJ, et al (2009) A cluster-randomised intervention trial against *Schistosoma japonicum* in the Peoples' Republic of China: bovine and human transmission. PLoS ONE 4: e5900. 19521532 10.1371/journal.pone.0005900 PMC2690852

[12] Gray DJ, Williams GM, Li Y, Chen H, Li RS, et al (2007) A cluster-randomized bovine intervention trial against *Schistosoma japonicum* in the People's Republic of China: design and baseline results. Am J Trop Med Hyg 77: 866–874. 17984344 PMC2756501

[13] Gray DJ, Williams GM, Li Y, McManus DP (2008) Transmission dynamics of *Schistosoma japonicum* in the lakes and marshlands of China. PLoS ONE 3: e4058. 19115007 10.1371/journal.pone.0004058 PMC2605259

[14] Gray DJ, Williams GM, Li YS, Chen HG, Forsyth S, et al (2009) The role of bovines in human *Schistosoma japonicum* infection in the People's Republic China. Am J Trop Med Hyg 81: 1046.

[15] Guo J, Li Y, Gray DJ, Hu G, Chen H, et al (2006) A drug-based intervention study on the importance of buffaloes for human *Schistosoma japonicum* infection around Poyang Lake, People's Republic of China. Am J Trop Med Hyg 74: 335–341. 16474093

[16] Guo J, Ross AG, Lin D, Williams GM, Chen H, et al (2001) A baseline study on the importance of bovines for human *Schistosoma japonicum* infection around Poyang Lake, China. Am J Trop Med Hyg 65: 272–278. 11693868 10.4269/ajtmh.2001.65.272

[17] Wu H, Qin Y, Chu K, Meng R, Liu Y, et al (2010) High prevalence of *Schistosoma japonicum* infection in water buffaloes in the Philippines assessed by real-time polymerase chain reaction. Am J Trop Med Hyg 82: 646–652. 20348514 10.4269/ajtmh.2010.09-0638 PMC2844580

[18] Cabrera BD (1976) Schistosomiasis japonica in field rats in Leyte, Philippines. Southeast Asian J Trop Med Public Health 7: 50–55. 1027108

[19] Carabin H, Balolong E, Joseph L, McGarvey ST, Johansen MV, et al (2005) Estimating sensitivity and specificity of a faecal examination method for *Schistosoma japonicum* infection in cats, dogs, water buffaloes, pigs, and rats in Western Samar and Sorsogon Provinces, The Philippines. Int J Parasitol 35: 1517–1524. 16188261 10.1016/j.ijpara.2005.06.010

[20] McGarvey ST, Carabin H, Balolong E Jr, Bélisle P, Fernandez T, et al (2006) Cross-sectional associations between intensity of animal and human infection with *Schistosoma japonicum* in Western Samar province, Philippines. Bull World Health Organ 84: 446–452. 16799728 10.2471/blt.05.026427 PMC2627378

[21] Yu JM, de Vlas SJ, Jiang QW, Gryseels B (2007) Comparison of the Kato-Katz technique, hatching test and indirect hemagglutination assay (IHA) for the diagnosis of *Schistosoma japonicum* infection in China. Parasitol Int 56: 45–49. 17188018 10.1016/j.parint.2006.11.002

[22] Gordon CA, Acosta LP, Gray DJ, Olveda R, Jarilla B, et al (2012) High prevalence of *Schistosoma japonicum* infection in carabao from Samar province, the Philippines: implications for transmission and control. PLoS Negl Trop Dis doi: 10.1371/journal.pntd.0001778 10.1371/journal.pntd.0001778 PMC3447974 23029571

[23] Willingham AL, Johansen MV, Barnes EH (1998) A new technique for counting *Schistosoma japonicum* eggs in pig faeces. Southeast Asian J Trop Med Public Health 29: 128–130. 9740285

[24] Cringoli G (2006) FLOTAC, a novel apparatus for a multivalent faecal egg count technique. Parassitologica 48: 381–384. 17176947

[25] Cringoli G, Rinaldi L, Maurelli MP, Utzinger J (2010) FLOTAC: new multivalent techniques for qualitative and quantitative copromicroscopic diagnosis of parasites in animals and humans. Nat Protoc 5: 503–515. 20203667 10.1038/nprot.2009.235

[26] Glinz D, Silué KD, Knopp S, Lohouringnon LK, Yao KP, et al (2010) Comparing diagnostic accuraccy of Kato-Katz, koga agar plate, ether-concentration, and FLOTAC for *Schistosoma mansoni* and soli-transmitted helminths. PLoS Negl Trop Dis 4: e754. 20651931 10.1371/journal.pntd.0000754 PMC2907416

[27] Knopp S, Glinz D, Rinaldi L, Mohammed KA, N'Goran EK, et al (2009) FLOTAC: a promising technique for detecting helminth eggs in human faeces. Trans R Soc Trop Med Hyg 103: 1190–1194. 19573886 10.1016/j.trstmh.2009.05.012

[28] Jones MK, Balen J (2007) Magnetic beads for schistosomiasis diagnosis. PLoS Negl Trop Dis 1: e159. 18160983 10.1371/journal.pntd.0000159 PMC2154394

[29] Teixeira CF, Neuhauss E, Ben R, Romanzini J, Graeff-Teixeira C (2007) Detection of *Schistosoma mansoni* eggs in feces through their interaction with paramagnetic beads in a magnetic field. PLoS Negl Trop Dis 1: e73. 18060086 10.1371/journal.pntd.0000073 PMC2100366

[30] Angeles JM, Goto Y, Kirinoki M, Asada M, Leonardo LR, et al (2012) Utilization of ELISA using thioredoxin peroxidase-1 and tandem repeat proteins for diagnosis of *Schistosoma japonicum* infection among water buffaloes. PLoS Negl Trop Dis 6. 10.1371/journal.pntd.0001800 PMC3429387 22953018

[31] Katz N, Chaves A, Pellegrino J (1972) A simple device for quantitative stool thick smear technique in schistosomiasis mansoni. Rev Inst Med Trop Sao Paulo 14: 397–400. 4675644

[32] Zhang Y-Y, Luo J-P, Liu Y-M, Wang Q-Z, Chen J-H, et al (2009) Evaluation of Kato-Katz examination method in three areas with low-level endemicity of schistosomiasis japonica in China: a Bayesian modeling approach. Acta Trop 112: 16–22. 19501562 10.1016/j.actatropica.2009.05.020

[33] Davis GM, Wu W, Chen H, Liu H, Guo J, et al (2002) A baseline study of the importance of bovines for human *Schistosoma japonicum* infections around Poyang Lake, China: Villages studied and snail sampling strategy. Am J Trop Med Hyg 66: 359–371. 12164289 10.4269/ajtmh.2002.66.359

[34] Wang T, Johansen MV, Zhang S, Wang F, Wu W, et al (2005) Transmission of *Schistosoma japonicum* by humans and domestic animals in the Yangtze River valley, Anhui province, China. Acta Trop 96: 198–204. 16188215 10.1016/j.actatropica.2005.07.017

[35] Zou FC, Dong GD, Yang JF, Xie YJ, Zhang YG, et al (2010) Prevalences of *Schistosoma japonicum* infection in reservoir hosts in south-western China. Ann Trop Med Parasitol 104: 181–185. 20406586 10.1179/136485910X12607012374118

[36] Su ZW, Hu CQ, Fu Y, Cheng W, Huang XB (1994) Role of several hosts in transmission of Schistosomiasis japonica in lake region. Chinese Journal of Parasitology and Parasitic Diseases 12: 48–51. 8044907

[37] Lu D, Wang T, Rudge JW, Donnely CA, Fang G, et al (2010) Contrasting reservoirs for Schistosoma japonicum between marshland and hilly regions in Anhui, China – a two-year longitudinal parasitological survey. Parasitology 137: 99–110. 19723358 10.1017/S003118200999103X

[38] Liang S, Yang C, Zhong B, Qiu D (2006) Re-emerging schistosomiasis in hilly and mountainout areas of Sichuan, China. Bull World Health Organ 84: 139–144. 16501732 10.2471/blt.05.025031 PMC2626530

[39] Balen J, Zhao ZY, Williams GM, McManus DP, Raso G, et al (2007) Prevalence, intensity and associated morbidity of *Schistosoma japonicum* infection in the Dongting Lake region, China. Bull World Health Organ 85: 519–526. 17768500 10.2471/BLT.06.034033 PMC2636368

[40] Fernandez TJ, Tarafder MR, Balolong E, Joseph L, Willingham AL III, et al (2007) Prevalence of *Schistosoma japonicum* infection among animals in fifty villages of Samar Province, the Philippines. Vector Borne Zoonotic Dis 7: 147–155. 17627431 10.1089/vbz.2006.0565

[41] Matsumoto J, Kirinoki M, Kawai S, Chigusa Y (1999) Prevalence of schistosomiasis japonica among school children and animal reservoirs in Oriental Mindoro, Philippines. Japanese Journal of Tropical Medicine and Hygiene 27: 175–180.

[42] Dumag PU (1981) Epidemiology of animal schistosomiasis in the Philippines. Phil J Anim Indust 36: 1–23.

[43] Pesigan TP, Farooq M, Hairston NG, Jauregui JJ, Garcia EG, et al (1958) Studies on *Schistosoma japonicum* infection in the Philippines. 1. General considerations and epidemiology. Bull World Health Organ 18: 345–455. 13536797 PMC2537660

[44] Fernandez TJ Jr, Petilla T, Banez B (1982) An epidemiological study on *Schistosoma japonicum* in domestic animals in Leyte, Philippines. Southeast Asian J Trop Med Public Health 13: 575–579. 7170639

