# Correction: A Novel Procedure for Precise Quantification of *Schistosoma japonicum* Eggs in Bovine Feces

**DOI:** 10.1371/annotation/2c375f5c-78e4-4920-9498-014bf8d2aff3

**Published:** 2012-12-19

**Authors:** Bin Xu, Catherine A. Gordon, Wei Hu, Donald P. McManus, Hong-Gen Chen, Darren J. Gray, Chuan Ju, Xiao-Jun Zeng, Geoffrey N. Gobert, Jun Ge, Wei-Ming Lan, Shu-Ying Xie, Wei-Sheng Jiang, Allen G. Ross, Luz P. Acosta, Remigio Olveda, Zheng Feng

The image for Figure 2 is incorrect. Please view the correct image here:

http://www.plosntd.org/corrections/pntd.0001885.g002.cn.tif

